# Discordant vascular parameter measurements in diabetic and non-diabetic eyes detected by different optical coherence tomography angiography devices

**DOI:** 10.1371/journal.pone.0234664

**Published:** 2020-06-16

**Authors:** Yi Chen, Sawarin Laotaweerungsawat, Tong Zhao, Zeeshan Haq, Xiuyun Liu, Catherine Psaras, Daphne Yang, Jay M. Stewart

**Affiliations:** 1 Department of Ophthalmology, University of California, San Francisco, CA, United States of America; 2 Department of Ophthalmology, Zuckerberg San Francisco General Hospital and Trauma Center, San Francisco, CA, United States of America; 3 Shenzhen Eye Hospital, Shenzhen Key Laboratory of Ophthalmology, Affiliated Shenzhen Eye Hospital of Jinan University, Shenzhen, China; 4 Department of Ophthalmology, Charoenkrung Pracharak Hospital, Bangkok, Thailand; 5 Department of Ophthalmology, China-Japan Friendship Hospital, Beijing, China; 6 Department of Physiological Nursing, University of California, San Francisco, CA, United States of America; Bascom Palmer Eye Institute, UNITED STATES

## Abstract

**Purpose:**

To compare quantitative changes in macular parameters in diabetic patients detected by two optical coherence tomography angiography (OCTA) instruments.

**Methods:**

80 phakic eyes were classified as no diabetes, diabetes without diabetic retinopathy (DR), mild non-proliferative diabetic retinopathy (NPDR), and severe NPDR or proliferative DR (PDR). OCTA was performed using devices from two manufacturers (Zeiss and Heidelberg). Superficial and deeper vascular skeleton density (SVSD, DVSD), superficial and deeper vessel area density (SVAD, DVAD), choriocapillaris flow voids (CCFV), and choroidal flow voids (CFV) were calculated. Inter-device comparisons were performed using the size comparison index (SCI) and the discrepancy index (DI).

**Results:**

The two devices were inconsistent in SVSD, DVSD, DVAD, CCFV and CFV parameters (all *P* < 0.05). In addition, the SCI was positive for DVAD (all P < 0.001) and negative for SVSD, DVSD, CCFV and CFV in all groups (all *P* <0.001), except for DVSD in severe NPDR or PDR. The discrepancy index was not significantly different among groups for SVD, SPD, DVD, DPD and CFV (all *P>* 0.05). The mean DI of CCFV was statistically different between the four groups (*P* < 0.001).

**Conclusions:**

The two instruments were largely inconsistent in the measurement of macular parameters relevant to DR. The choice of imaging device can impact OCTA analytics and should be taken into account when drawing conclusions about DR-related changes.

## Introduction

Diabetes mellitus (DM) is a chronic metabolic disease that causes end-organ complications, including diabetic retinopathy (DR). DR is a microvascular disease that has profound effects on the retinal vasculature and is rapidly becoming more prevalent worldwide.[1] In the past, fluorescein angiography (FA) was the gold standard for studying DR.[2] However, due to the inconvenience and various potential risks of FA, optical coherence tomography (OCTA) is now increasingly used in DR research.

OCTA can produce a high-resolution blood flow image of all vessel layers within the retina in a rapid, non-invasive manner. OCTA can also analyze choroidal blood flow by detecting flow voids.[3] Two widely used OCTA instruments on the market today, Heidelberg Engineering’s SPECTRALIS® and Zeiss’s CIRRUS™ HD-OCT, utilize different algorithms for OCTA image acquisition. Heidelberg uses a full spectrum amplitude decorrelation algorithm while Zeiss uses optical microangiography.[4]

Several publications have discussed the measurement differences between OCTA machines. However, there are several limitations to prior reports in this area. These studies did not correct for the impact of axial length on the resulting OCTA image. Previous literature has suggested that a change in axial length (AL) causes an OCTA image magnification error that affects the results.[5] In addition, previous comparative studies only accounted for retinal perfusion parameters and did not evaluate choroidal perfusion. Recent literature has highlighted the importance of changes in choroidal blood flow in patients with diabetic retinopathy.[6–8] Therefore, a comprehensive evaluation of OCTA imaging in DR should consider the choroid in addition to the retina.

The purpose of the present study was to evaluate and compare quantitative changes in macular perfusion parameters in diabetic patients using two spectral domain (SD) OCTA instruments. To make the comparison as meaningful and broadly applicable as possible, both diabetic patients with various stages of DR and non-diabetic patients were included. And, in order to improve upon prior studies, both retinal and choroidal parameters were evaluated, and axial length corrections were applied to all images.

## Methods

### Subjects

This study was approved by the Human Research Protection Program (HRPP) at the University of California, San Francisco (UCSF). The UCSF HRPP granted a waiver of consent, affirming that patient welfare would not be adversely affected by doing so. All research adhered to the tenets of the Declaration of Helsinki. Patients diagnosed with DR for the first time and diabetic patients without DR seen in the Department of Ophthalmology at Zuckerberg San Francisco General Hospital and Trauma Center from May to July 2019 were included. Similar aged subjects without either DM or any form of retinopathy were also recruited as a control group. The right eye from each patient was included, and if the right eye image was of poor quality, the left eye was used.

Exclusion criteria consisted of (1) patients with type 1 DM and DR secondary to type 1 DM; (2) any history of ocular injury, ocular surgery including cataract surgery, and other vitreoretinal diseases except DR; (3) DR patients with a treatment history of laser, intravitreal injection or vitrectomy; (4) or low quality OCTA images indicated by the presence of significant motion artifacts, defocus or blur, or signal strength less than 6 (Zeiss) or 30 dB (Heidelberg).

The relevant demographic and clinical information for all subjects including gender, age, hypertension status, and the most recent hemoglobin A1c (HbA1c) were recorded. AL was measured with the IOL master 700 (Carl Zeiss Meditec, Dublin, CA, USA). Ultra-widefield fundus images (Optos Daytona, Optos Plc, Dunfermline, United Kingdom) were obtained for each patient, and DR severity was graded by the department's reading center affiliated with its telemedicine DR-screening program. Patients were divided into four groups according to the severity of their DR: non-diabetic (control group), DM without DR, mild non-proliferative diabetic retinopathy (NPDR), and severe NPDR or proliferative diabetic retinopathy (PDR).

### Image acquisition and processing

OCTA images (pixels 421 x 421) were obtained using a Cirrus high-definition–OCT instrument (Model 5000, Carl Zeiss Meditec Inc.) with a scan area of 3 x 3 mm centered at the fovea. The enhanced depth imaging (EDI) mode was used. Both eyes of each participant were imaged with the AngioPlex software at a wavelength of 840 nm and a speed of 68,000 per second. Motion-related artifacts were minimized with the help of the tracking algorithm on the Cirrus device. The superficial retinal layer (SRL) of the retinal OCTA image was segmented with an inner boundary set at the ILM and an outer boundary set at the outer border of the inner plexiform layer (IPL). Per Yasukura et al, we defined the IPL layer as 70% of the retinal thickness, from the ILM to OPL.[9] The deeper retinal layer (DRL) OCTA image was auto-segmented with an inner boundary set at the outer border of the IPL and an outer boundary set at the outer border of the RPE—110 um. The choriocapillaris layer of the retinal OCTA image was segmented with an inner boundary set at the RPE + 29 um and an outer boundary set at the outer border of the RPE + 49 um. The choroidal layer of the retinal OCTA image was segmented with an inner boundary set at the outer border of the RPE + 64 um and an outer boundary set at the outer border of the RPE + 115 um.[9] The magnification factor of the image was corrected according to a previously reported formula [5,10,11]: Dt^2^/Dm^2^ = 0.002066*(AL-1.82)^2^.

OCTA images (pixels 512 x 512) were also obtained for the same patient during the same session using a SPECTRALIS^®^ OCT Angiography Module (Spectralis OCT2, Heidelberg Engineering, Heidelberg, Germany) with a scan of 10° x 10° centered at the fovea. These dimensions are equivalent to the 3×3 mm areas used in the Zeiss machine scans. The EDI mode was used. Both eyes of each participant were imaged with the HEYEX software at a wavelength of 870 nm and a speed of 85,000 per second. Motion-related artifacts were minimized with the tracking algorithm on the Spectralis device. The superficial layer of the retinal OCTA image was auto-segmented with an inner boundary set at the NFL and an outer boundary set at the outer border of the IPL—17 um. The deep layer of the retinal OCTA image was segmented with an inner boundary set at the outer border of the IPL + 22 um and an outer boundary set at the outer border of the OPL. The choriocapillaris layer of the retinal OCTA image was segmented with an inner boundary set at Bruch’s membrane (BM) and an outer boundary set at the outer border of the BM + 20 um. The choroidal layer of the retinal OCTA image was segmented with an inner boundary set at the outer border of the BM + 20 um and an outer boundary set at the outer border of the BM + 100 um.[12,13] The magnification factor of the image was corrected[5,14] by first calculating the C-curve value, then using the HEYEX software to calculate the magnification: C-curve = 301.76/[1333/(AL-1.83)-K-21.76], with K representing the patient’s corneal curvature in diopters.

**Fig 1:**
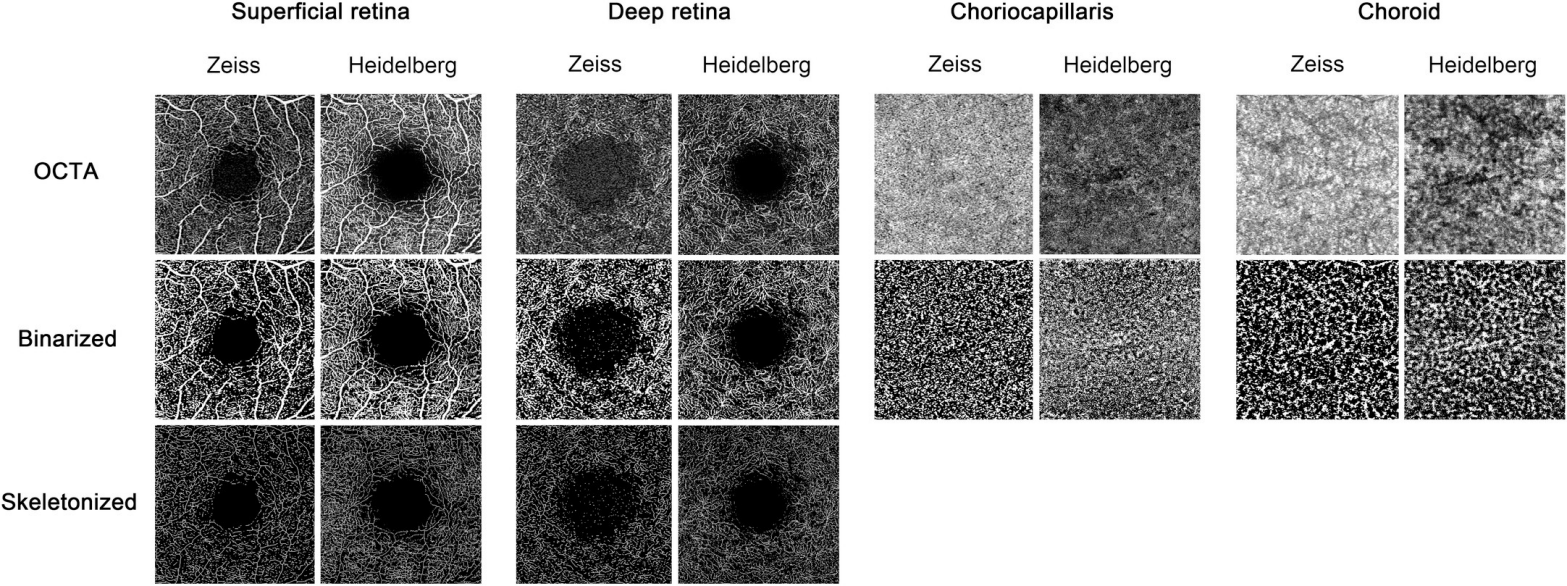
Comparison of images acquired from Zeiss and Heidelberg devices. Optical coherence tomography angiography (OCTA) images, binarized images, and skeletonized images of the superficial and deep retina, choriocapillaris, and choroid using two different OCTA devices to examine a 46-year-old woman with diabetes and no DR.

All images of each layer were analyzed with ImageJ software (1.8.0_112, http://imagej.nih.gov/ij/; National Institutes of Health, Bethesda, Maryland, USA) to acquire the superficial vascular skeleton density (SVSD), superficial vessel area density (SVAD), deep vessel skeleton density (DVSD), deep vessel area density (DVAD), choriocapillaris flow voids (CCFV), and choroidal flow voids (CFV). Vascular skeleton density (VSD) or vessel length density (VLD) was defined as the total length of skeletonized perfused vasculature per unit area.[15,16] Vessel area density (VAD) was defined as the total area of perfused vasculature per unit area.[15,17] Flow voids (FV) were defined as the total area of perfused per unit resolvable area without flow signal.[18–20] All image stacks were converted to binary images using the software's auto-threshold feature and the noise was removed from the generated dual derivative image (Fig 1). To simplify the comparison of OCTA parameter values between machines, we utilized a size comparison index (SCI), calculated via the formula SCI = Zeiss/Heidelberg—1, to estimate the difference in size of the above parameters within each disease group. With this calculation, if the SCI is positive, the value of a particular parameter obtained from the Zeiss device is greater than that obtained with the Heidelberg device. If the SCI is negative, the value from the Zeiss device is less than that of the Heidelberg device. In a similar way, we used a discrepancy index (DI), calculated via the formula DI = | (Zeiss/Heidelberg) - 1|, to assess the discrepancy of the above parameters between the disease groups.

### Statistical analysis

Statistical analysis was performed using SPSS version 24 (IBM, New York, USA) and MedCalc Statistical Software version 19.0.6 (MedCalc Software bvba, Ostend, Belgium; https://www.medcalc.org; 2019). Data are generally presented as mean ± standard deviation. All data were found to fit a normal distribution by the Kolmogorov-Smirnov test. Consistency of measurements between the two OCTA imaging devices was evaluated by Bland-Altman plots.[21–24] A one sample T test was used to assess whether mean SCI values were significantly different from zero. Then we used the sign of the mean to compare the size. One-way ANOVA using DI values was used to analyze whether there was a significant difference in OCTA parameters between at least two of the four patient groups. If a significant difference was found, post-hoc tests were used to identify specific significant pairwise comparisons. For all statistical tests, a p-value of less than 0.05 was considered to be statistically significant with a Bonferroni correction applied when appropriate.

## Results

A total of 80 eyes of 80 subjects were included in the study. The mean age was 56.75 ±10.01 years with a range from 34 to 80. Thirty-seven (46.25%) subjects were female. Excluding non-diabetic subjects, the mean HbA1c of diabetic patients was 7.90 ± 1.88%. All diabetic subjects had type 2 diabetes. Thirty-two subjects (40%) had hypertension. The mean axial length was 23.29 ± 0.87 mm. There were 20 eyes in the non-diabetic control group (group 1), 20 eyes with DM without DR (group 2), 20 with mild NPDR (group 3) and 20 with severe NPDR or PDR (group 4). There were no significant differences between the four groups with regards to age, gender, hypertension or axial length. A significant difference in HbA1c was present among the three groups (P = 0.003), and HbA1c was positively correlated with grouping (r = 0.483, P < 0.001). Detailed demographics are summarized in Table 1.

**Table 1:** Demographic and clinical characteristics of study subjects.

	Group 1	Group 2	Group 3	Group 4	P value
Subjects, n	20	20	20	20	
Eyes, n	20	20	20	20	
Age, y, mean ± SD	54.6 ± 9.2	55.7 ± 10.0	57.5 ± 12.0	59.2 ± 8.6	0.492
Sex-female, n (%)	7 (35%)	9 (45%)	10 (50%)	11 (55%)	0.637
Hypertension					0.417
Yes, n (%)	5 (25%)	8 (40%)	9 (45%)	10 (50%)	
No, n (%)	15 (75%)	12 (60%)	11 (55%)	10 (50%)	
HbA1c, mean ± SD	N/A	6.9 ± 1.2	7.8 ± 1.9	9.0 ± 1.9	0.003
Missing, n (%)	N/A	4 (20%)	3 (15%)	4 (20%)	
Axial length, mm, mean ± SD	23.62 ± 1.06	23.24 ± 0.67	23.25 ± 0.83	23.04 ± 0.82	0.186

Group 1: nondiabetic controls; Group 2: diabetes without DR; Group 3: mild NPDR; Group 4: severe NPDR or PDR.

### Within-group OCTA parameter measurement comparisons

**Fig 2:**
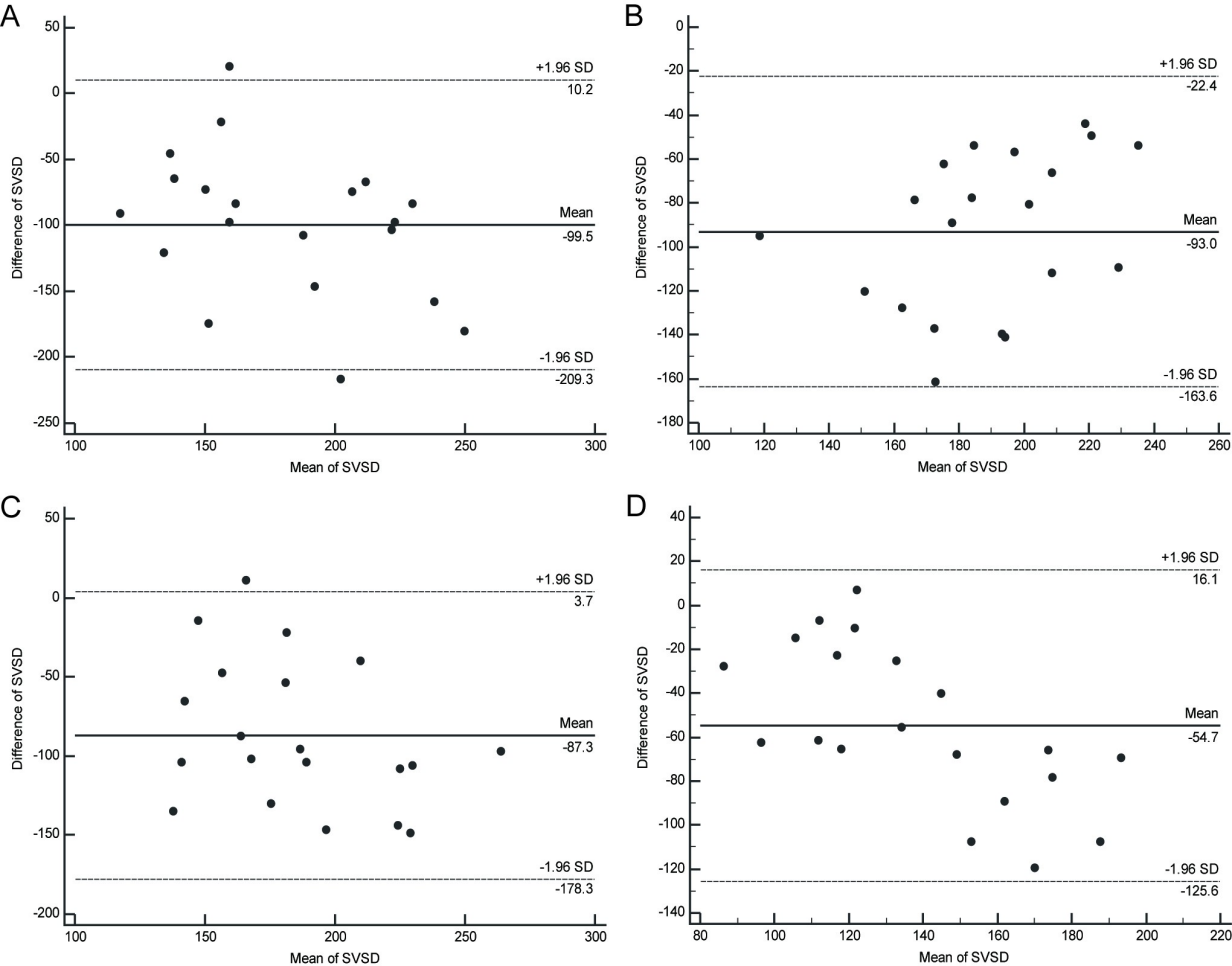
Zeiss and Heidelberg comparison using the SVSD parameter in each diabetic retinopathy severity group. Bland-Altman plots. Inconsistency between Zeiss and Heidelberg in all four diabetic retinopathy severity groups: (A) Group 1, (B) Group 2, (C) Group 3, (D) Group 4. The solid line indicates the mean of the differences; the upper and lower dotted lines indicate the upper and lower limits of agreement (LA). Group 1: nondiabetic controls; Group 2: diabetes without DR; Group 3: mild NPDR; Group 4: severe NPDR and PDR.

**Fig 3:**
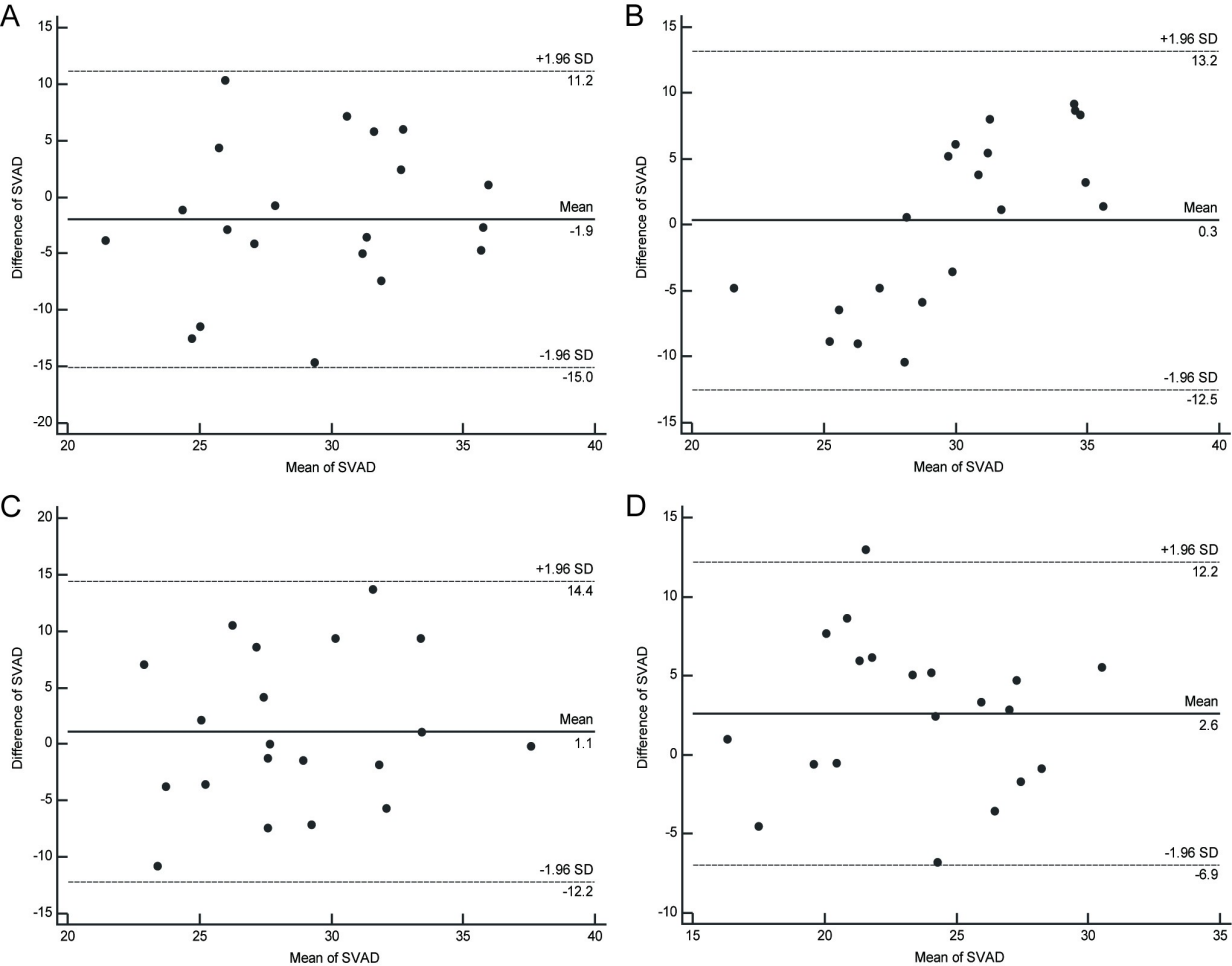
Zeiss and Heidelberg comparison using the SVAD parameter in each diabetic retinopathy severity group. Bland-Altman plots. A: Zeiss and Heidelberg are consistent in Group 1. B: Zeiss and Heidelberg are consistent in Group 2. C: Zeiss and Heidelberg are consistent in Group 3. D: Zeiss and Heidelberg are inconsistent in Group 4. The solid line indicates the mean of the differences; the upper and lower dotted lines indicate the upper and lower limits of agreement (LA). Group 1: nondiabetic controls; Group 2: diabetes without DR; Group 3: mild NPDR; Group 4: severe NPDR and PDR.

Detailed results of the Bland-Altman analyses are shown in Table 2. Across all four groups, Zeiss and Heidelberg OCTAs were inconsistent in SVSD, DVSD, DVAD, CCFV and CFV parameters. Across groups 1–3, Zeiss and Heidelberg OCTA were partially consistent in SVAD parameters. Fig 2 shows Bland-Altman plots of SVSD measured with Zeiss and Heidelberg in groups 1–4, indicating poor agreement between the two instruments and the possibility of a systematic error yielding unreliable results. In contrast, Fig 3 shows Bland-Altman plots of SVAD measured with Zeiss and Heidelberg in groups 1–4, indicating good agreement between the two instruments in groups 1–3 and poor agreement between the two instruments in group 4, with values in all four plots showing a mean value not significantly different from zero, with random scatter that is contained within acceptable limits, confirming the reliability of the comparison. For a comparison of DVSD, DVAD, CCFV and CFV parameters, please refer to S1–S4 Figs.

**Table 2:** Results of Bland-Altman analysis for VSD, VAD, CCFV and CFV for comparisons between methods A (Zeiss) and B (Heidelberg).

Parameter	Difference Mean	*P* (H_0_: Mean = 0)	Lower LA (95% CI)	Upper LA (95% CI)
Group 1				
SVSD	-99.5	<0.001	-209.3	10.2
SVAD	-1.9	0.212	-15.0	11.2
DVSD	-65.7	<0.001	-148.3	16.9
DVAD	8.1	<0.001	-3.0	19.1
CCFV	-20.6	<0.001	-32.7	-8.5
CFV	-8.3	<0.001	-21.8	5.3
Group 2				
SVSD	-93.0	<0.001	-163.6	-22.4
SVAD	0.3	0.817	-12.5	13.2
DVSD	-50.1	<0.001	-131.5	31.3
DVAD	8.0	<0.001	0.5	15.4
CCFV	-20.2	<0.001	-30.1	-10.4
CFV	-9.2	<0.001	-21.9	3.5
Group 3				
SVSD	-87.3	<0.001	-178.3	3.7
SVAD	1.1	0.484	-12.2	14.4
DVSD	-63.8	<0.001	-125.7	-2.0
DVAD	8.5	<0.001	-1.3	18.4
CCFV	-17.4	<0.001	-28.8	-6.0
CFV	-8.1	<0.001	-22.1	6.0
Group 4				
SVSD	-54.7	<0.001	-125.6	16.1
SVAD	2.6	0.027	-6.9	12.2
DVSD	15.8	0.028	-73.8	42.3
DVAD	6.5	<0.001	-7.3	20.2
CCFV	-16.2	<0.001	-27.3	-5.2
CFV	-11.6	<0.001	-21.0	-2.2

SVSD: superficial vessel skeleton density; SVAD: superficial vessel area density; DVSD: deep vessel skeleton density; DVAD: deep vessel area density; CCFV: choriocapillaris flow voids; CFV: choroidal flow voids; SCI: size comparison index; LA: limits of agreement.

Group 1: nondiabetic controls; Group 2: diabetes without DR; Group 3: mild NPDR; Group 4: severe NPDR and PDR.

### Within-group OCTA parameter magnitude comparisons

Across all four groups, the SCI was negative for SVSD, DVSD, CCFV and CFV parameters. Therefore, the value of Zeiss OCTA parameters was less than on the Heidelberg OCTA, with the difference reaching statistical significance (all P<0.001, except for DVSD in Group 4). Across all four groups, the SCI was positive for DVAD parameters. Therefore, the value of DVAD on the Zeiss OCTA was significantly greater than that on the Heidelberg OCTA (all P < 0.001).

In group 1, the SCI was negative for the SVAD parameter, but the difference did not reach statistical significance (P *>* 0.05). In groups 2 and 3, the SCI was positive for the SVAD parameter, but the difference was not significant (*P >*0.05). In group 4, the SCI was positive for the SVAD parameter (*P*< 0.05). Between the four disease groups, the direction of the difference (either positive or negative) was consistent across both machines for all parameters except SVAD.

Detailed results of the magnitude of parameter measurements are shown in Table 3.

**Table 3:** SCIs of OCTA parameters within DR severity stage groups.

	Group 1		Group 2		Group 3		Group 4	
	SCI Mean*	*P*	SCI Mean*	*P*	SCI Mean*	*P*	SCI Mean*	*P*
SVSD	-0.41	<0.001	-0.40	<0.001	-0.36	<0.001	-0.30	<0.001
SVAD	-0.04	0.397	0.01	0.771	0.07	0.284	0.15	0.021
DVSD	-0.30	<0.001	-0.23	<0.001	-0.31	<0.001	-0.07	0.287
DVAD	0.53	<0.001	0.47	<0.001	0.56	<0.001	0.45	0.001
CCFV	-0.39	<0.001	-0.37	<0.001	-0.31	<0.001	-0.25	<0.001
CFV	-0.19	<0.001	-0.21	<0.001	-0.17	<0.001	-0.24	<0.001

*Comparisons were always performed considering the difference between method B (Heidelberg) and method A (Zeiss). Thus, a positive bias means Zeiss values are greater than Heidelberg.

Group 1: nondiabetic controls; Group 2: diabetes without DR; Group 3: mild NPDR; Group 4: severe NPDR or PDR.

### Between-group OCTA parameter comparisons

**Fig 4:**
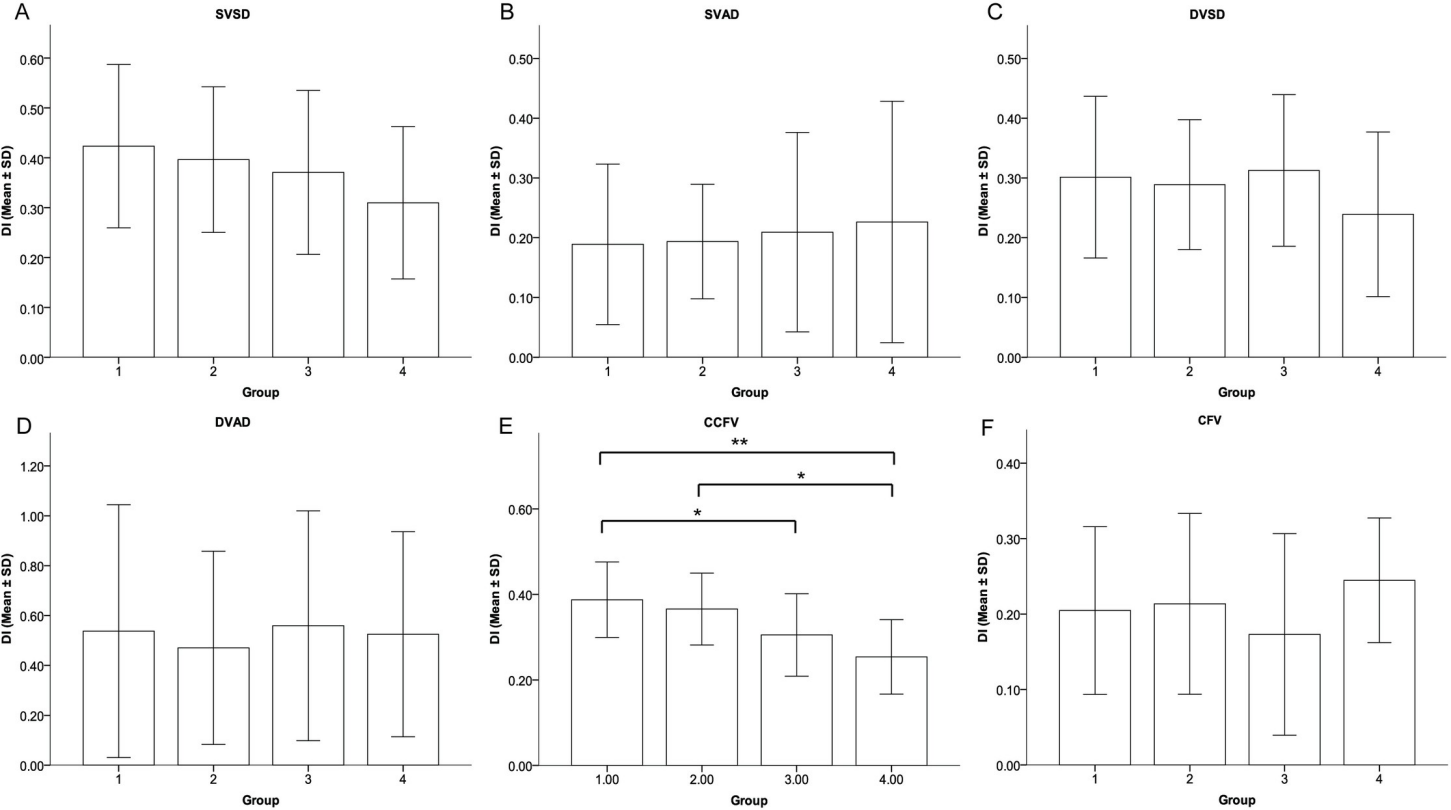
Comparison of DIs for all OCTA parameters between diabetic retinopathy severity groups. A, B, C, D, F: there was no statistically significant difference between the groups. E: the DI of the CCFV in Group 4 was significantly smaller than in Groups 1 and 2, and the DI in Group 3 was significantly smaller than in Group 1. * P < 0.05 and ** P < 0.001. Group 1: nondiabetic controls; Group 2: diabetes without DR; Group 3: mild NPDR; Group 4: severe NPDR and PDR. Bars represent means, and whiskers represent standard deviations.

Comparing between the four groups, the DI was not significantly different in the SVD, SPD, DVD, DPD and CFV parameters (all *P >* 0.05). The mean DI of CCFV was statistically different between all 4 groups (*P* < 0.001). The mean DI of the CCFV decreased from group 1 to group 4 as disease severity increased (*P* < 0.001). The mean DI of the CCFV in group 3 was significantly less than in group 1 (*P* = 0.027). The mean DI of the CCFV in Group 4 was significantly larger than group 2 or 3 (*P* < 0.001 in both cases). Detailed comparison results are shown in Fig 4 and Table 4.

**Table 4:** Comparisons of DIs of OCTA parameters between diabetic retinopathy severity stage groups.

	DI (Mean ± SD)				*P* value					
	Group 1	Group 2	Group 3	Group 4	4 VS 3	4 VS 2	4 VS 1	3 VS 2	3 VS 1	2 VS 1
SVSD	0.42±0.16	0.40±0.15	0.37±0.16	0.31±0.15	1	0.509	0.149	1	1	1
SVAD	0.19±0.13	0.19±0.10	0.21±0.17	0.23±0.20	1	1	1	1	1	1
DVSD	0.30±0.14	0.29±0.11	0.31±0.13	0.24±0.14	0.436	1	0.764	1	1	1
DVAD	0.54±0.51	0.47±0.39	0.56±0.46	0.53±0.41	1	1	1	1	1	1
CCFV	0.39±0.09	0.37±0.08	0.31±0.10	0.25±0.09	0.431	0.001	<0.001	0.208	0.027	1
CFV	0.20±0.11	0.21±0.12	0.17±0.13	0.24±0.08	0.294	1	1	1	0.884	1

Group 1: nondiabetic controls; Group 2: diabetes without DR; Group 3: mild NPDR; Group 4: severe NPDR and PDR.

## Discussion

The use of OCTA is becoming more widespread, especially in patients with DR.[25,26] As such, it is important to know whether different commercially available devices will provide similar information about retinal and choroidal perfusion in patients with various levels of microvascular disease. This study examined the retinal vasculature in the eyes of nondiabetic individuals and patients with diabetes with and without DR using SD-OCTA images from two different OCTA devices. We compared the results of Zeiss and Heidelberg OCTA scans acquired under as similar conditions as possible. The impact of disease severity on differences was assessed.

We found that all Zeiss and Heidelberg results, in both diabetic and nondiabetic populations, were inconsistent in the SVSD, DVSD, DVAD, CCFV and CFV parameters. Corvi et al previously reported a similar result analyzing only nondiabetic patients.[22] In our study, we found that differences between the two machines exist for diabetic patients as well. Possible reasons for the differences include inconsistent segmentation and differing algorithms used by the two machines.[4] In most cases, these two devices may not be interchangeable in the examination of either nondiabetic or diabetic patients. Therefore, studies reporting OCTA parameters should be interpreted in the context of which device was used to perform the scans. We also found that SVAD parameter results from both machines were consistent in nondiabetic individuals and patients with no or mild diabetic retinopathy. The inconsistency of SVAD results relative to the other parameters bears further investigation in future studies.

In this study, we used the SCI to compare the values obtained by the two machines. In the case of using two parameters of VSD (SVSD, DVSD) and FV (CCFV, CFV), the Heidelberg OCTA yielded larger values than Zeiss. The direction of this difference was consistent across all four disease groups. A larger VSD value from the same patient scan is likely due to more microvascular length information being captured.[15,16] This suggests that capillary resolution is greater with Heidelberg than with Zeiss when considering the entire vascular complex as a whole, including both superficial and deep components. Among the DVAD parameters, values obtained with the Heidelberg device were less than those from Zeiss, and the direction of the difference was consistent across all four disease groups. Larger DVAD values likely reflect more information about the length and diameter of a blood vessel being obtained.[15,17] However, the DVSD results showed higher values from the Heidelberg machine. This suggests that the Zeiss device is only potentially superior in vessel diameter resolution in the deep layer, for unknown reasons.[27] Lastly, the larger the FV value, the larger the captured area with no signal. Our results indicate that the area of the no-flow signal captured by the Heidelberg device is larger than that of the Zeiss device. At present, SD-OCTA cannot completely remove the projection of the retinal layer onto the choroid layer. We speculate that Heidelberg, which has a higher blood flow signal value in the retinal layer, has more projected occlusion signals in the CC layer. So the FV value is higher.

This is the first study to report the use of DI parameters to quantify the differences between two OCTA machines in different disease groups. In the retina and choroid layer, the DI did not differ between the four disease groups. This may be due to the impact of layering and algorithms on DI, which exceeds the impact of disease changes. However, in the choriocapillaris layer, we found a trend that the differences between the two machines decreased as DR disease severity increased. In the choriocapillaris layer, the default stratification of the two machines is similar,[9,12,13] and the difference may be caused by different algorithms.[28,29] Despite attempts to minimize the effect of artifacts by excluding poor-quality scans, image quality (potentially associated with media opacity) may have affected our measurements, particularly in the severe NDPR or PDR group. [30,31] An increase in retinal thickness associated with worsening diabetic retinopathy may affect the acquisition of choriocapillaris layer images. Nonetheless, we suspect the effect of reduced image quality likely exceeds the effect of different algorithms, resulting in the difference between the two machines being correspondingly reduced.

Different OCTA machines have different stratification definitions, and many parameters that are commonly used to report retinal and choroidal blood flow are not uniform. It can therefore be very difficult to make comparisons between different machines, and contradictory results have been reported in the literature. Munk et al found that there was no difference in the overall vessel density among four different devices using OCTA.[32] On the other hand, Mihailovic et al reported that the average area value of the FAZ was significantly different between all instrument pairs (Optovue, Canon and Heidelberg).[21] In addition, Corvi et al found that VD and FAZ measurements were different for both the superficial and deep capillary plexuses between seven different OCTA devices.[22]

In light of these varying results, we took several steps in our analysis to try to ensure the accuracy of the comparison between the two systems. First, we corrected for the effect of axial length on the magnification of OCTA images, a step that has not been consistently reported in prior literature. Second, we used the same software and imaging processing method to analyze the images obtained from the two machines. As such, we believe it is reasonable to attribute the disparate results generated by the devices to different imaging and processing algorithms rather than characteristics of the eyes themselves; additionally, the presence of these differences in both nondiabetic and diabetic eyes confirms that the results are not disease-dependent.

One limitation of this study is that multiple OCTA devices are currently commercially available, but our analysis only compares two of them. Additional insight may be gained by incorporating analyses of DR patients using other machines. Another limitation is that due to sample size constraints, we did not include a group with moderate NPDR; however, since the studied groups included patients with both mild and severe disease, it is likely that the majority of disease-related differences in performance between the two devices were able to be identified. In addition, there was no assessment of the precision of the two devices in the form of measuring the repeatability of measurements from the same patient on the same device. Finally, we did not use swept-source (SS)-OCTA to evaluate choroidal vessel flow. SS-OCT, which utilizes a longer wavelength than spectral domain devices, can theoretically penetrate deeper than SD-OCT. However, both machines have been reported to have difficulty visualizing choroidal vessels due to the overlying RPE.[33] Given these limitations, we elected to use FV as a proxy to evaluate choroidal blood flow since it does not require clear visualization of vessels. [3,19]

## Conclusion

This novel study explored the comparison of two OCTA machines after correcting for the effects of axial length. Ultimately, we found that the Zeiss and Heidelberg machines were inconsistent with respect to several OCTA parameters. Our results suggest that the Heidelberg machine may have superior capillary resolution in both the superficial and deep retinal layers. However, the Zeiss machine may have superior vessel diameter resolution in the deep retina layer. Lastly, we found that inter-machine choriocapillaris layer measurement differences were negatively associated with diabetic retinopathy severity.

## Supporting information

S1 Fig Zeiss and Heidelberg comparison using the DVSD parameter in each diabetic retinopathy severity group. Bland-Altman plots. A: Zeiss and Heidelberg are inconsistent in Group 1. B: Zeiss and Heidelberg are inconsistent in Group 2. C: Zeiss and Heidelberg are inconsistent in Group 3. D: Zeiss and Heidelberg are inconsistent in Group 4. The solid line indicates the mean of the differences; the upper and lower dotted lines indicate the upper and lower limits of agreement (LA).
(JPG)

S2 Fig Zeiss and Heidelberg comparison using the DVAD parameter in each diabetic retinopathy severity group. Bland-Altman plots. A: Zeiss and Heidelberg are consistent in Group 1. B: Zeiss and Heidelberg are consistent in Group 2. C: Zeiss and Heidelberg are consistent in Group 3. D: Zeiss and Heidelberg are inconsistent in Group 4. The solid line indicates the mean of the differences; the upper and lower dotted lines indicate the upper and lower limits of agreement (LA).
(JPG)

S3 Fig Zeiss and Heidelberg comparison using the CCFV parameter in each diabetic retinopathy severity group. Bland-Altman plots. A: Zeiss and Heidelberg are inconsistent in Group 1. B: Zeiss and Heidelberg are inconsistent in Group 2. C: Zeiss and Heidelberg are inconsistent in Group 3. D: Zeiss and Heidelberg are inconsistent in Group 4. The solid line indicates the mean of the differences; the upper and lower dotted lines indicate the upper and lower limits of agreement (LA).
(JPG)

S4 Fig Zeiss and Heidelberg comparison using the CFV parameter in each diabetic retinopathy severity group. Bland-Altman plots. A: Zeiss and Heidelberg are consistent in Group 1. B: Zeiss and Heidelberg are consistent in Group 2. C: Zeiss and Heidelberg are consistent in Group 3. D: Zeiss and Heidelberg are inconsistent in Group 4. The solid line indicates the mean of the differences; the upper and lower dotted lines indicate the upper and lower limits of agreement (LA).
(JPG)

## References

[1] Stitt AW, Curtis TM, Chen M, et al The progress in understanding and treatment of diabetic retinopathy. Progress in Retinal and Eye Research. 2016;51:156–186. 10.1016/j.preteyeres.2015.08.001 26297071

[2] Classification of diabetic retinopathy from fluorescein angiograms: ETDRS report number 11. Ophthalmology. 1991;98(5, Supplement):807–822. 2062514

[3] Spaide RF, Fujimoto JG, Waheed NK. Optical coherence tomography angiography. Retina (Philadelphia, Pa.). 2015;35(11):2161–2162. https://www.ncbi.nlm.nih.gov/pubmed/26502006. 10.1097/IAE.0000000000000881 26502006 PMC4710360

[4] Zhang Q, Zhang A, Chen C, Wang RK. Methods and algorithms for optical coherence tomography-based angiography: A review and comparison. Journal of Biomedical Optics. 2015;20(10):100901 10.1117/1.JBO.20.10.100901 26473588 PMC4881033

[5] Sampson DM, Gong P, An D, et al Axial length variation impacts on superficial retinal vessel density and foveal avascular zone area measurements using optical coherence tomography angiography. Investigative ophthalmology & visual science. 2017;58(7):3065–3072. https://www.ncbi.nlm.nih.gov/pubmed/28622398. 10.1167/iovs.17-21551 28622398

[6] Melancia D, Vicente A, Cunha J, Abegão Pinto L, Ferreira J. Diabetic choroidopathy: A review of the current literature. Graefes Arch Clin Exp Ophthalmol. 2016;254(8):1453–1461. https://www.ncbi.nlm.nih.gov/pubmed/27109344. 10.1007/s00417-016-3360-8 27109344

[7] Coscas G, Lupidi M, Coscas F, Chhablani J, Cagini C. Optical coherence tomography angiography in healthy subjects and diabetic patients. Ophthalmologica. Journal international d'ophtalmologie. International journal of ophthalmology. Zeitschrift fur Augenheilkunde. 2018;239(2–3):61–73. https://www.ncbi.nlm.nih.gov/pubmed/29268269. 10.1159/000485323 29268269

[8] Conti FF, Qin VL, Rodrigues EB, et al Choriocapillaris and retinal vascular plexus density of diabetic eyes using split-spectrum amplitude decorrelation spectral-domain optical coherence tomography angiography. The British journal of ophthalmology. 2019;103(4):452–456. https://www.ncbi.nlm.nih.gov/pubmed/29793926. 10.1136/bjophthalmol-2018-311903 29793926

[9] Yasukura S, Murakami T, Suzuma K, et al Diabetic nonperfused areas in macular and extramacular regions on wide-field optical coherence tomography angiography. Investigative ophthalmology & visual science. 2018;59(15):5893–5903. https://www.ncbi.nlm.nih.gov/pubmed/30550612. 10.1167/iovs.18-25108 30550612

[10] Bennett AG, Rudnicka AR, Edgar DF. Improvements on littmann's method of determining the size of retinal features by fundus photography. Graefe's archive for clinical and experimental ophthalmology = Albrecht von Graefes Archiv fur klinische und experimentelle Ophthalmologie. 1994;232(6):361–367. https://www.ncbi.nlm.nih.gov/pubmed/8082844. 10.1007/BF00175988 8082844

[11] Leung CK, Cheng ACK, Chong KKL, et al Optic disc measurements in myopia with optical coherence tomography and confocal scanning laser ophthalmoscopy. Investigative Ophthalmology & Visual Science. 2007;48(7):3178–3183. http://www.iovs.org/cgi/content/abstract/48/7/3178. 10.1167/iovs.06-1315 17591887

[12] Campbell JP, Zhang M, Hwang TS, et al Detailed vascular anatomy of the human retina by projection-resolved optical coherence tomography angiography. Scientific reports. 2017;7(1):42201 https://www.ncbi.nlm.nih.gov/pubmed/28186181. 10.1038/srep42201 28186181 PMC5301488

[13] Rocholz Roland, PhD; Michel M. Teussink, PhD; Rosa Dolz-Marco, PhD; Claudia Holzhey; Jan F. Dechent, PhD; Ali Tafreshi; et al SPECTRALIS optical coherence tomography angiography (OCTA): Principles and clinical applications. Heidelberg Engineering Academy, accessed September 23, 2019.

[14] Delori F, Greenberg JP, Woods RL, et al Quantitative measurements of autofluorescence with the scanning laser ophthalmoscope. Investigative ophthalmology & visual science. 2011;52(13):9379–9390. https://www.ncbi.nlm.nih.gov/pubmed/22016060. 10.1167/iovs.11-8319 22016060 PMC3250263

[15] Chu Z, Lin J, Gao C, et al Quantitative assessment of the retinal microvasculature using optical coherence tomography angiography. Journal of Biomedical Optics. 2016;21(6):066008 10.1117/1.JBO.21.6.066008 27286188 PMC4901200

[16] Agemy SA, Scripsema NK, Shah CM, et al Retinal vascular perfusion density mapping using optical coherence tomography angiography in normals and diabetic retinopathy patients. Retina. 2015;35(11):2353–2363. 10.1097/IAE.0000000000000862 26465617

[17] Reif Roberto, Qin Jia, An Lin, Zhi Zhongwei, Dziennis Suzan, Wang Ruikang. Quantifying optical microangiography images obtained from a spectral domain optical coherence tomography system. International journal of biomedical imaging. 2012;2012:509783–11. 10.1155/2012/509783 22792084 PMC3389716

[18] Rochepeau C, Kodjikian L, Garcia M, et al Optical coherence tomography angiography quantitative assessment of choriocapillaris blood flow in central serous chorioretinopathy. American Journal of Ophthalmology. 2018;194:26–34. https://www.sciencedirect.com/science/article/pii/S0002939418303854. 10.1016/j.ajo.2018.07.004 30053475

[19] Spaide RF MD. Choriocapillaris flow features follow a power law distribution: Implications for characterization and mechanisms of disease progression. American Journal of Ophthalmology. 2016;170:58–67. https://www.clinicalkey.es/playcontent/1-s2.0-S0002939416303695. 10.1016/j.ajo.2016.07.023 27496785

[20] Zhang Q, Zheng F, Motulsky EH, et al A novel strategy for quantifying choriocapillaris flow voids using swept-source OCT angiography. Investigative ophthalmology & visual science. 2018;59(1):203–211. https://www.ncbi.nlm.nih.gov/pubmed/29340648. 10.1167/iovs.17-22953 29340648 PMC5770182

[21] Mihailovic N, Brand C, Lahme L, et al Repeatability, reproducibility and agreement of foveal avascular zone measurements using three different optical coherence tomography angiography devices. PloS one. 2018;13(10):e0206045 https://www.ncbi.nlm.nih.gov/pubmed/30335839. 10.1371/journal.pone.0206045 30335839 PMC6193722

[22] Corvi F, Pellegrini M, Erba S, Cozzi M, Staurenghi G, Giani A. Reproducibility of vessel density, fractal dimension, and foveal avascular zone using 7 different optical coherence tomography angiography devices. American Journal of Ophthalmology. 2018;186:25–31. https://www.sciencedirect.com/science/article/pii/S0002939417304890. 10.1016/j.ajo.2017.11.011 29169882

[23] Giavarina D. Understanding bland altman analysis. Biochemia medica. 2015;25(2):141–151. https://www.ncbi.nlm.nih.gov/pubmed/26110027. 10.11613/BM.2015.015 26110027 PMC4470095

[24] Vujosevic S, Toma C, Villani E, et al Early detection of microvascular changes in patients with diabetes mellitus without and with diabetic retinopathy: Comparison between different swept-source OCT-A instruments. Journal of Diabetes Research. 2019;2019:1–12. https://search.proquest.com/docview/2253825711. 10.1155/2019/2547216 31281849 PMC6594252

[25] Ishibazawa A, Nagaoka T, Takahashi A, et al Optical coherence tomography angiography in diabetic retinopathy: A prospective pilot study. American Journal of Ophthalmology. 2015;160(1):3–44.e1. https://www.clinicalkey.es/playcontent/1-s2.0-S000293941500224X. 10.1016/j.ajo.2015.04.021 25896459

[26] Durbin MK, An L, Shemonski ND, et al Quantification of retinal microvascular density in optical coherence tomographic angiography images in diabetic retinopathy. JAMA Ophthalmology. 2017;135(4):370–376. 10.1001/jamaophthalmol.2017.0080 28301651 PMC5470403

[27] Kim AY, Chu Z, Shahidzadeh A, Wang RK, Puliafito CA, Kashani AH. Quantifying microvascular density and morphology in diabetic retinopathy using spectral-domain optical coherence tomography angiography. Investigative ophthalmology & visual science. 2016;57(9):OCT362–OCT370. https://www.ncbi.nlm.nih.gov/pubmed/27409494. 10.1167/iovs.15-18904 27409494 PMC4968771

[28] Lauermann JL, Heiduschka P, Nelis P, et al Comparison of choriocapillaris flow measurements between two optical coherence tomography angiography devices. Ophthalmologica. Journal international d'ophtalmologie. International journal of ophthalmology. Zeitschrift fur Augenheilkunde. 2017;237(4):238–246. https://www.ncbi.nlm.nih.gov/pubmed/28433988. 10.1159/000464355 28433988

[29] Lauermann JL, Eter N, Alten F. Optical coherence tomography angiography offers new insights into choriocapillaris perfusion. Ophthalmologica. Journal international d'ophtalmologie. International journal of ophthalmology. Zeitschrift fur Augenheilkunde. 2018;239(2–3):74–84. https://www.ncbi.nlm.nih.gov/pubmed/29353272. 10.1159/000485261 29353272

[30] Nesper PL, Roberts PK, Onishi AC, et al Quantifying microvascular abnormalities with increasing severity of diabetic retinopathy using optical coherence tomography angiography. Investigative ophthalmology & visual science. 2017;58(6):BIO307-BIO315. https://www.ncbi.nlm.nih.gov/pubmed/29059262. 10.1167/iovs.17-21787 29059262 PMC5693005

[31] Ting DSW, Tan GSW, Agrawal R, et al Optical coherence tomographic angiography in type 2 diabetes and diabetic retinopathy. JAMA Ophthalmology. 2017;135(4):306–312. 10.1001/jamaophthalmol.2016.5877 28208170

[32] Munk MR, Giannakaki-Zimmermann H, Berger L, et al OCT-angiography: A qualitative and quantitative comparison of 4 OCT-A devices. PloS one. 2017;12(5):e0177059 https://www.ncbi.nlm.nih.gov/pubmed/28489918. 10.1371/journal.pone.0177059 28489918 PMC5425250

[33] Diaz JD, Wang JC, Oellers P, et al Imaging the deep choroidal vasculature using spectral domain and swept source optical coherence tomography angiography. Journal of VitreoRetinal Diseases. 2018;2(3):146–154. https://journals.sagepub.com/doi/full/10.1177/2474126418771805. 29930992 10.1177/2474126418771805 PMC6007996

