# Supplementary figures and images for: Discordant vascular parameter measurements in diabetic and non-diabetic eyes detected by different optical coherence tomography angiography devices
Source: PLoS One. 2020 Jun 16;15(6):e0234664. doi: 10.1371/journal.pone.0234664 (PMC7297376; doi:10.1371/journal.pone.0234664)

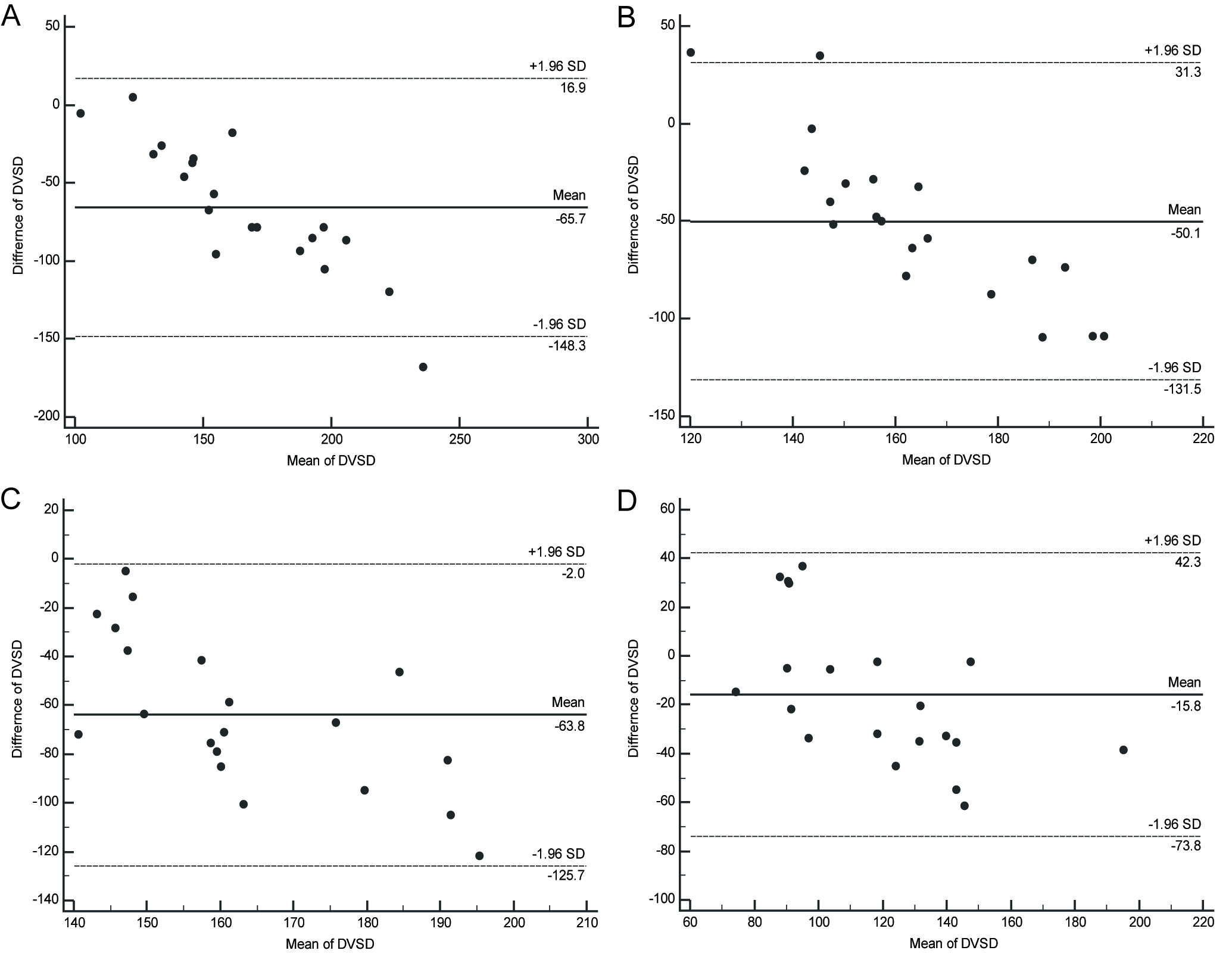

Supplement: S1 Fig — Bland-Altman plots. A: Zeiss and Heidelberg are inconsistent in Group 1. B: Zeiss and Heidelberg are inconsistent in Group 2. C: Zeiss and Heidelberg are inconsistent in Group 3. D: Zeiss and Heidelberg are inconsistent in Group 4. The solid line indicates the mean of the differences; the upper and lower dotted lines indicate the upper and lower limits of agreement (LA). (JPG) [file pone.0234664.s001.jpg]

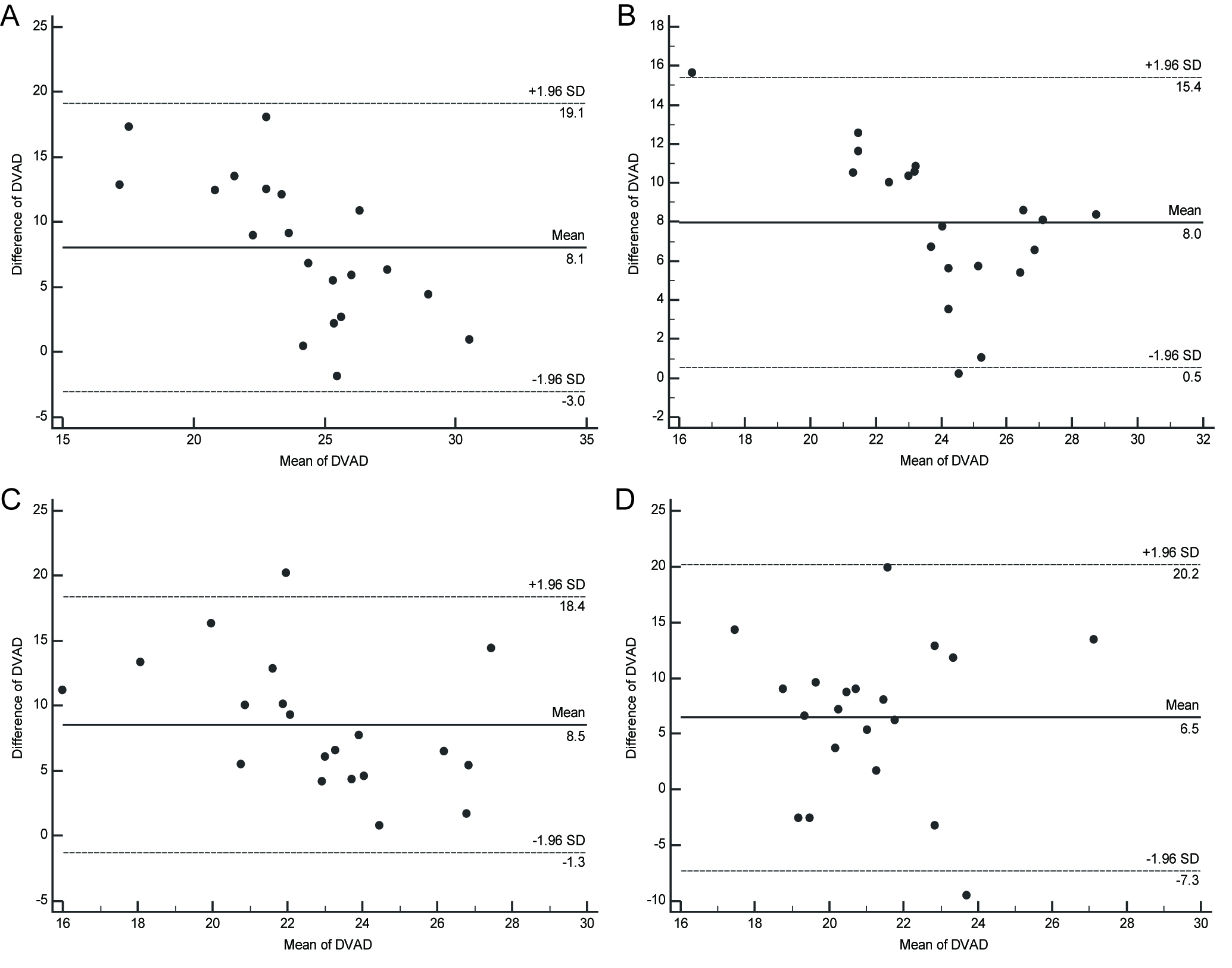

Supplement: S2 Fig — Bland-Altman plots. A: Zeiss and Heidelberg are consistent in Group 1. B: Zeiss and Heidelberg are consistent in Group 2. C: Zeiss and Heidelberg are consistent in Group 3. D: Zeiss and Heidelberg are inconsistent in Group 4. The solid line indicates the mean of the differences; the upper and lower dotted lines indicate the upper and lower limits of agreement (LA). (JPG) [file pone.0234664.s002.jpg]

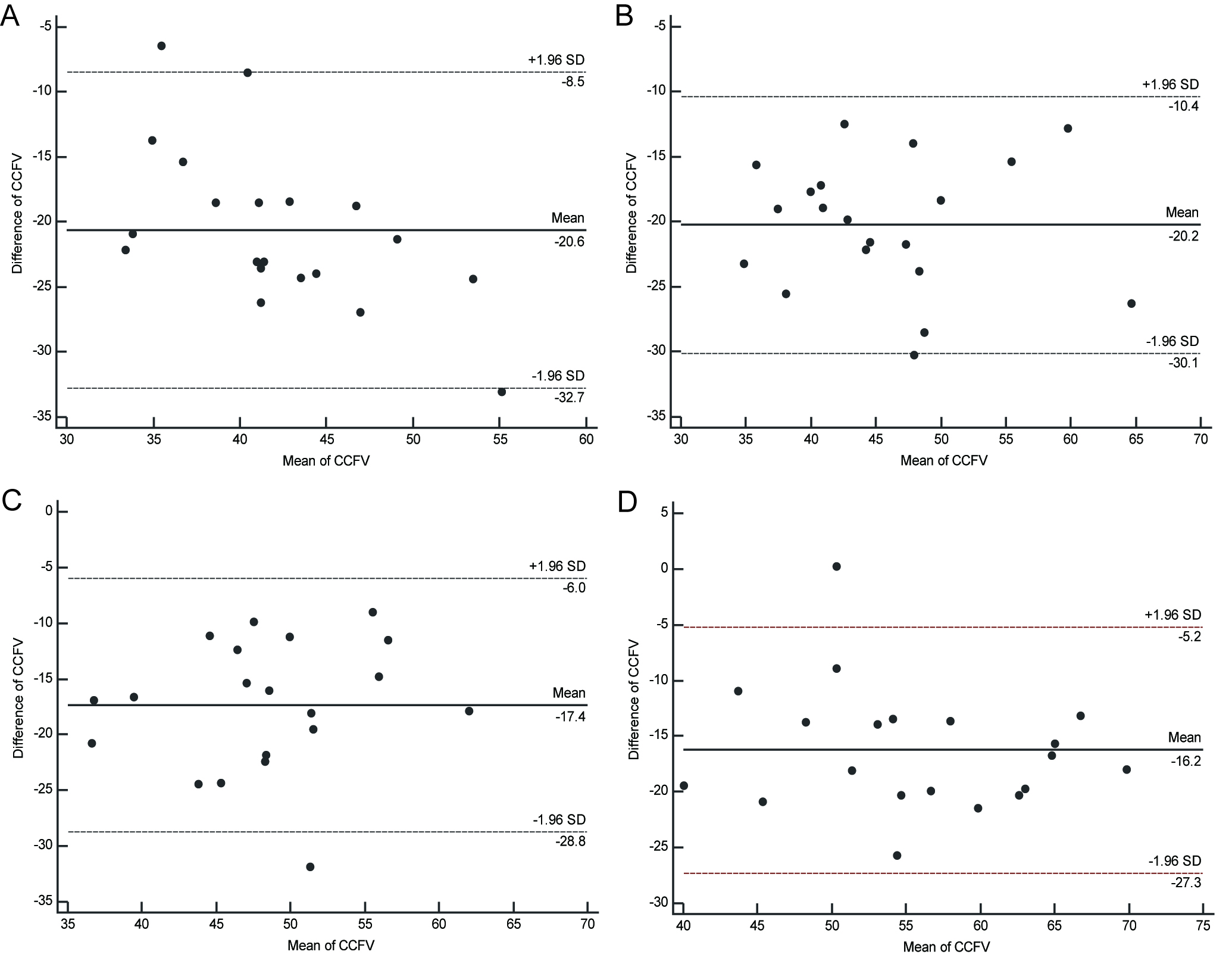

Supplement: S3 Fig — Bland-Altman plots. A: Zeiss and Heidelberg are inconsistent in Group 1. B: Zeiss and Heidelberg are inconsistent in Group 2. C: Zeiss and Heidelberg are inconsistent in Group 3. D: Zeiss and Heidelberg are inconsistent in Group 4. The solid line indicates the mean of the differences; the upper and lower dotted lines indicate the upper and lower limits of agreement (LA). (JPG) [file pone.0234664.s003.jpg]

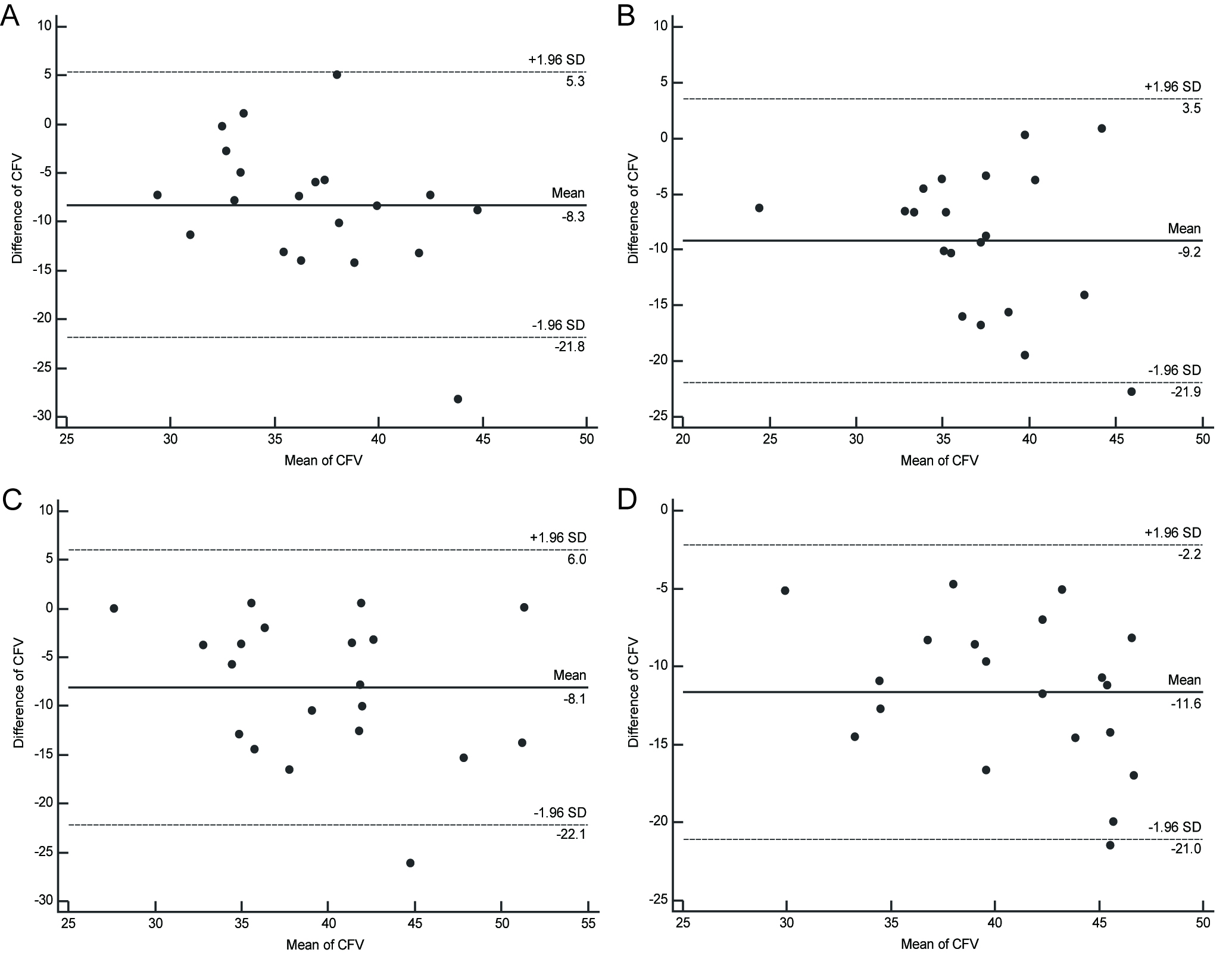

Supplement: S4 Fig — Bland-Altman plots. A: Zeiss and Heidelberg are consistent in Group 1. B: Zeiss and Heidelberg are consistent in Group 2. C: Zeiss and Heidelberg are consistent in Group 3. D: Zeiss and Heidelberg are inconsistent in Group 4. The solid line indicates the mean of the differences; the upper and lower dotted lines indicate the upper and lower limits of agreement (LA). (JPG) [file pone.0234664.s004.jpg]
